# The key role of glutamine for protein expression and isotopic labeling in insect cells

**DOI:** 10.1016/j.jbc.2023.105142

**Published:** 2023-08-06

**Authors:** Feng-Jie Wu, Domenic Kronenberg, Ines Hertel, Stephan Grzesiek

**Affiliations:** Biozentrum, University of Basel, Basel, Switzerland

**Keywords:** glutamine, isotope labeling, tricarboxylic acid cycle, insect cells, NMR, G protein-coupled receptor, Abelson kinase, green fluorescent protein

## Abstract

Nuclear magnetic resonance studies of many physiologically important proteins have long been impeded by the necessity to express such proteins in isotope-labeled form in higher eukaryotic cells and the concomitant high costs of providing isotope-labeled amino acids in the growth medium. Economical routes use isotope-labeled yeast or algae extracts but still require expensive isotope-labeled glutamine. Here, we have systematically quantified the effect of ^15^N_2_-glutamine on the expression and isotope labeling of different proteins in insect cells. Sufficient levels of glutamine in the medium increase the protein expression by four to five times relative to deprived conditions. ^1^H-^15^N nuclear magnetic resonance spectroscopy shows that the ^15^N atoms from ^15^N_2_-glutamine are scrambled with surprisingly high (60–70%) efficiency into the three amino acids alanine, aspartate, and glutamate. This phenomenon gives direct evidence that the high energy demand of insect cells during baculovirus infection and concomitant heterologous protein expression is predominantly satisfied by glutamine feeding the tricarboxylic acid cycle. To overcome the high costs of supplementing isotope-labeled glutamine, we have developed a robust method for the large-scale synthesis of ^15^N_2_-glutamine and partially deuterated ^15^N_2_-glutamine-α,β,β-d_3_ from inexpensive precursors. An application is shown for the effective large-scale expression of the isotope-labeled β_1_-adrenergic receptor using the synthesized ^15^N_2_-glutamine-α,β,β-d_3_.

The observation of magnetically active atomic nuclei in a protein by nuclear magnetic resonance (NMR) can provide invaluable atomistic insights into protein function that is not accessible in static crystallographic or cryo-EM structures. Such studies usually require incorporation of the stable isotopes ^15^N, ^13^C, and ^2^H. This can be achieved for *E. coli* expression by growth on inexpensive isotope-labeled ammonium, glucose, and ^2^H_2_O. However, many physiologically important proteins, such as membrane receptors of human origin, cannot easily be expressed in *E. coli*, but need the more advanced protein folding, post-translational modification, and membrane insertion machineries of higher eukaryotes. The latter expression hosts require the supplementation of very expensive isotope-labeled amino acids to the growth media (1, 2, 3, 4, 5, 6, 7, 8, 9, 10, 11, 12, 13, 14).

To lower the costs, methods for supplementing isotope-labeled amino acids *via* lysed isotope-labeled yeast or algae extracts have been developed for insect and mammalian cells (1, 4, 9, 15). However, in such extracts, the amino acid glutamine is present only in very low amounts or completely absent due to hydrolysis during their preparation (4, 9). Glutamine is a crucial amino acid for insect or mammalian cell proliferation and survival (4, 16, 17, 18, 19, 20). The glutamine metabolism in cells produces α-ketoglutarate as an intermediate, which partakes in the tricarboxylic acid (TCA) cycle in mitochondria to produce energy (19, 21, 22, 23). Furthermore, glutamine is also the nitrogen donor for the biosynthesis of other nonessential amino acids, nucleotides, and glutathione (23). Thus, isotope-labeled glutamine needs to be added to the insect or mammalian cell media along with the yeast or algae extract to achieve high-efficiency isotope labeling and protein expression (4, 9). Commercially available glutamine is extremely expensive, in particular in deuterated form.

In this work, we have precisely quantified the effect of glutamine on recombinant protein expression and isotope labeling in the baculovirus insect cell (*Spodoptera frugiperda*, *Sf9*) expression system. The expression yield strongly correlates with the concentration of glutamine supplemented in the medium. The ^15^N atoms from supplemented ^15^N_2_-labeled glutamine account for more than 10% of all nitrogen atoms incorporated into the expressed proteins. Besides glutamine, the ^15^N atoms are transferred to three further amino acids, glutamate, aspartate, and alanine, with surprisingly high efficiency (60–70%). To make supplementation of isotope-labeled glutamine affordable, we have developed a robust method for the large-scale combined chemical and enzymatic synthesis of glutamine from inexpensive precursors. The method achieves effective synthesis of α,β-deuterated, ^15^N-labeled glutamine (^15^N_2_-glutamine-α,β,β-d_3_) as well as of protonated ^15^N_2_-glutamine at costs of ∼100 Euro/g. As an application, we show the high-yield production of the selectively (Ala, Asp, Glu, and Gln) ^15^N-labeled and partially deuterated β_1_-adrenergic receptor (β_1_AR).

## Results

### Glutamine is essential for high protein expression and isotopic labeling in insect cells

**Figure 1:**
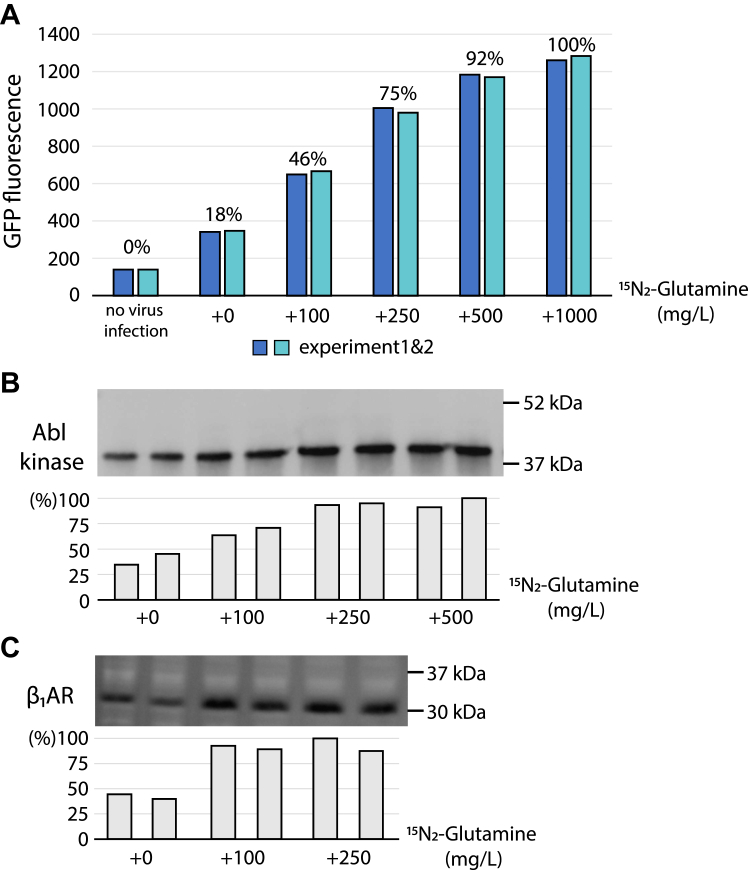
^**15**^**N**_**2**_**-glutamine concentration dependence of protein expression levels in insect cells.***A*, GFP. *B,* Abl kinase. *C*, β_1_AR. Protein expression levels were quantified by fluorescence for GFP and by anti-His Western blot analysis for Abl kinase and β_1_AR, respectively. The positions of molecular weight markers are indicated on the Western blots. β1AR, β1-adrenergic receptor; Abl, Abelson kinase; GFP, green fluorescent protein.

Previously, we reported that supplementation of 1 g/l glutamine to a yeast extract–based insect cell growth medium strongly improved the expression level of the Abelson kinase (Abl) protein (9). We have now extensively quantified this effect of the supplementation of ^15^N_2_-glutamine on the expression level of three proteins, the green fluorescent protein (GFP), Abl, and the turkey β_1_AR (Fig. 1, *A*–*C*). The increase of supplemented ^15^N_2_-glutamine monotonously increases the yield for all three tested proteins by 3- to 5-fold until a certain maximum level.

Importantly, but not surprisingly, the ^15^N_2_-glutamine amount required for reaching maximal expression depends on the overall expression yield of the protein of interest. Thus, maximal expression (∼40 mg/l) is achieved for the highly expressed GFP upon supplementation of 500 to 1000 mg/l ^15^N_2_-glutamine (Fig. 1*A*). In contrast, 250 to 500 mg/l ^15^N_2_-glutamine are required to achieve maximal expression (∼10–15 mg/l) for Abl kinase (Fig. 1*B*). For β_1_AR with the lowest expression yield (<1 mg/l), maximal expression is reached already when supplementing only ∼100 mg/l ^15^N_2_-glutamine (Fig. 1*C*). These results may provide guidance for other proteins on the minimally required glutamine amount to achieve maximal expression.

To understand how much ^15^N_2_-glutamine contributes to the total ^15^N labeling in proteins expressed in insect cells, we determined the ^15^N incorporation for GFP and Abl by mass spectrometry. For this, both proteins were expressed in a medium containing 8 g/l ^15^N uniformly labeled yeast extract supplemented with 1 g/l of either ^14^N_2_- or ^15^N_2_-glutamine. Under the best labeling conditions with ^15^N_2_-glutamine, about 86% of all GFP or Abl nitrogen atoms have ^15^N nuclei (Table S1). The missing 14% are caused by isotope dilution from the unlabeled cell culture and the virus. The use of ^14^N_2_-glutamine reduces the ^15^N content to about 76%, indicating that about 10% of ^15^N nuclei stem from the added glutamine (Table S1).

### Strong scrambling of ^15^N from glutamine to alanine, aspartate, and glutamate

**Figure 2:**
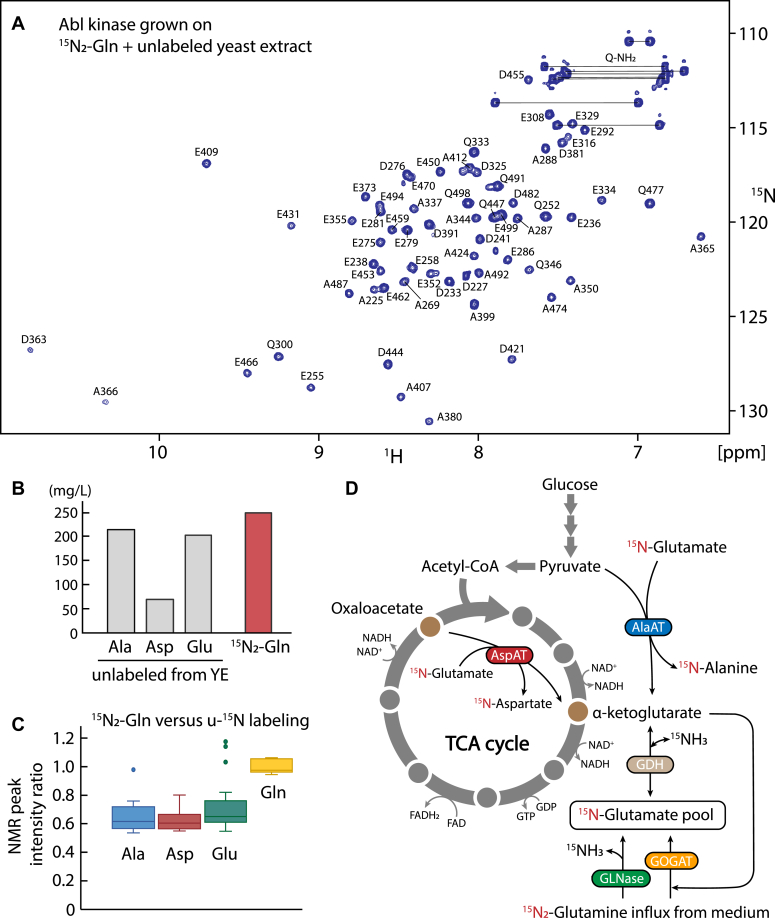
**Scrambling of**^**15**^**N atoms from**^**15**^**N**_**2**_**-glutamine to other amino acids in insect cells during protein expression induced by baculovirus infection.***A*, ^1^H-^15^N HSQC of Abl kinase expressed in insect cell medium containing 250 mg/l ^15^N_2_-glutamine and 8 g/l unlabeled yeast extract. Resonances are labeled with assignment information. *B*, concentrations of unlabeled alanine, aspartate, glutamate, and labeled ^15^N_2_-glutamine in the insect cell medium as established by HPLC-mass spectrometry (2, 9). *C*, quantification of ^15^N scrambling from ^15^N_2_-glutamine into alanine, aspartate, glutamate derived from the NMR peak intensities in spectra of ^15^N_2_-glutamine-labeled and uniformly ^15^N-labeled Abl kinase (see text). *D*, schematic diagram of glutaminolysis and energy production in the TCA cycle. The coupled reactions lead to the scrambling of ^15^N atoms from ^15^N_2_-glutamine to glutamate, alanine, and aspartate in insect cells after baculovirus infection. Abl, Abelson kinase; AlaAT, alanine transaminase; AspAT, aspartate transaminase; GDH, glutamate dehydrogenase; GLNase, glutaminase; GOGAT, glutamate synthase; HSQC, heteronuclear single quantum coherence spectrum.

As the nitrogen atoms from glutamine constitute only about 4% of all nitrogen atoms in both GFP and Abl, more than half of the nitrogen from the added ^15^N_2_-glutamine must have been transferred to other amino acids by the metabolism of the insect cells. The 2D ^1^H-^15^N heteronuclear single quantum coherence spectrum (HSQC) of Abl produced in insect cells with a medium containing 250 mg/l ^15^N_2_-glutamine and 8 g/l unlabeled yeast extract shows many strong resonances (Fig. 2*A*) that could be assigned using the published Abl kinase assignments (13). These resonances correspond to four types of amino acids, namely alanine, aspartate, glutamate, and glutamine, indicating that the ^15^N atoms from ^15^N_2_-glutamine are scrambled across these amino acids. At the noise level of the HSQC (Fig. S1), only five additional clear cross peaks could be detected arising from two asparagine residues with very intense backbone and sidechain resonances. A quantitative comparison to an HSQC of uniformly ^15^N-labeled Abl, produced using 95% ^15^N-labeled yeast extract with 0.25 g/l ^15^N_2_-glutamine supplementation (Fig. S2), indicates that the ^15^N incorporation of these asparagine residues as well as that of all other detectable amino acids besides alanine, aspartate, glutamate, and glutamine must be below 5%. To exclude ^15^N scrambling to prolines, which are not detected in the HSQC, we also analyzed hydrolyzed Abl by amino acid–specific HPLC mass spectrometry (2, 9) (Fig. S3), showing that the additional proline ^15^N incorporation is below 0.2%. An unlikely scrambling within the yeast extract itself was excluded by a further control incubating 250 mg/l ^15^N_2_-glutamine with 8 g/l unlabeled yeast extract at 27 °C for 24 h, which revealed no increase in the mass of alanine, aspartate, and glutamate within a limit of <0.5% (Fig. S4).

It is remarkable that ^15^N scrambling from ^15^N_2_-glutamine to proline, serine, cysteine, glycine, asparagine, lysine, and arginine is apparently very inefficient, albeit it has been reported that these amino acids can be synthesized during glutamine metabolism (23). The alanine, aspartate, and glutamate resonance intensities resulting from the ^15^N scrambling are almost as strong as the glutamine resonances (Fig. 2*A*). This is surprising considering that unlabeled alanine, aspartate, and glutamate and ^15^N-labeled glutamine were present in the medium at comparable concentrations (Fig. 2*B*) as established by amino acid-specific HPLC mass spectrometry (2, 9). To quantify the scrambling to the other amino acids, we calculated the intensity ratios of individual, nonoverlapping ^1^H-^15^N resonances in the HSQC of Fig. 2*A* and of the same resonances of the uniformly ^15^N-labeled Abl sample (Fig. S2). We then normalized these ratios by the ratio of the average glutamine peak intensities in both spectra. The obtained normalized intensity ratios indicate an average ^15^N-labeling of 63 to 67% for alanine, aspartate, and glutamate relative to glutamine (Fig. 2*C*), corroborating the very strong transfer from the glutamine ^15^N atoms to the latter amino acids, even against the large reservoir of their unlabeled forms in the medium. We attribute this phenomenon to the energy production by glutaminolysis in insect cells after baculovirus infection for protein expression (Fig. 2*D* and see below).

### Chemical and enzymatic reactions to economically synthesize ^15^N_2_-glutamine-α,β,β-d_3_

Obviously, the availability of sufficient amounts of glutamine in the insect cell medium is indispensable to achieve high protein expression yields. The quantitative scrambling data show that the supplemented glutamine must be isotope-labeled, if high levels of labeling shall be achieved not only for glutamine, but also for alanine, aspartate, and glutamate. Otherwise, unlabeled glutamine will scramble into these amino acids and dilute their labeling.

**Figure 3:**
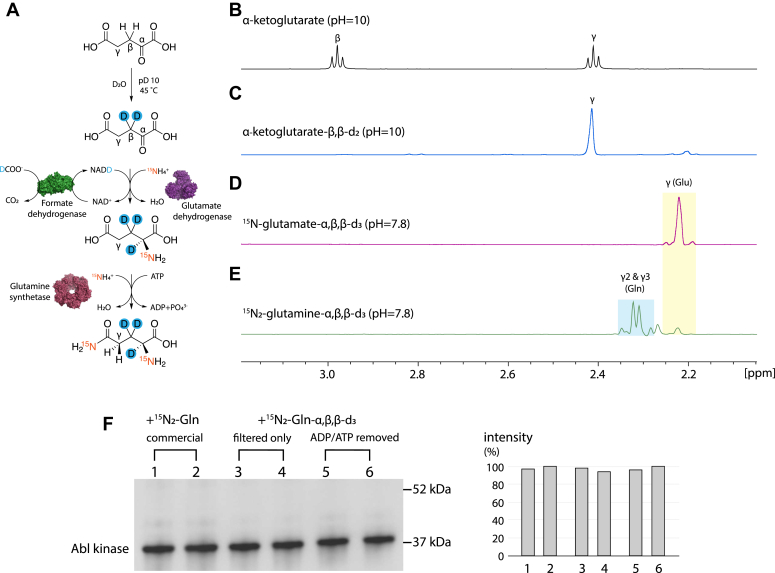
**Procedure for the inexpensive synthesis of isotope-labeled glutamine and quality control of reaction products.***A*, sequential reaction schemes for the combined chemical and enzymatic synthesis of ^15^N_2_-glutamine-α,β,β-d_3_ from inexpensive precursors. *B*–*E*, ^1^H NMR spectra for the quality control of the reaction products shown in (*A*). *F*, test of insect cell expression of Abl kinase using commercial ^15^N_2_-glutamine or ^15^N_2_-glutamine-α,β,β-d_3_ synthesized according to scheme (*A*). The expression efficiency was quantified by anti-His Western blot analysis (see Supplementary Experimental Procedures). The positions of molecular weight markers are indicated on the Western blots. The synthesized ^15^N_2_-glutamine-α,β,β-d_3_ was tested either as a filtered-only product or after ADP/ATP had been removed by ion-exchange beads (see text). Abl, Abelson kinase; NMR, nuclear magnetic resonance.

The costs of isotope-labeled glutamine may then become a major hurdle, since, *e.g.*, the current price for the least expensive, commercially available ^15^N_2_-glutamine is more than 1500 Euro per gram. This is particularly grave for proteins with low expression yields, such as G protein-coupled receptors, which often require several liters of labeled growth medium to obtain a single NMR sample. Based on methods to enzymatically synthesize ^15^N-glutamate from α-ketoglutarate (24) and ^15^N_2_-glutamine from ^15^N-glutamate (4), we have worked out here a robust procedure for the economical, large-scale production of ^15^N_2_-glutamine deuterated at the α, β hydrogen positions (^15^N_2_-glutamine-α,β,β-d_3_, Fig. 3). By omitting deuteration, also protonated ^15^N_2_-glutamine can be produced at even lower costs. Detailed protocols for the required procedures are documented in Table S2.

The first step of the ^15^N_2_-glutamine-α,β,β-d_3_ synthesis is a base-catalyzed proton-deuterium exchange reaction of the precursor α-ketoglutarate, which deuterates its two C^β^ hydrogens based on keto-enol tautomerism (25) (Fig. 3, *A*–*C*). The obtained, partially deuterated α-ketoglutarate-β,β-d_2_ is then lyophilized. The efficiency of the deuteration is >99.9% (determined from ^1^H NMR) with a recovery of 78% of the initial α-ketoglutarate.

As a next step, α-ketoglutarate, formate, and ammonium are enzymatically converted to glutamate by glutamate and formate dehydrogenases using the coenzyme NADH (Fig. 3*A*). The glutamate dehydrogenase (GDH) transfers a ^15^N-amide from ^15^N-ammonium and a hydrogen from NADH to the C^α^ position of α-ketoglutarate to form ^15^N-glutamate (26). The formate dehydrogenase (FDH) regenerates NADH from NAD^+^ by the transfer of a hydride from formate, which is converted to CO_2_ (27). In this dual enzymatic reaction, the H^α^ atom of glutamate originates from formate, and the deuteration of this hydrogen should be achievable using deuterated formate. Indeed, carrying out this reaction in H_2_O with α-ketoglutarate-β,β-d_2_, ^15^N-ammonium, and deuterated formate produced ^15^N-glutamate-α,β,β-d_3_, which was fully (>99.95%) deuterated in the α and β positions and only protonated at the γ position as shown by its ^1^H NMR spectrum (Fig. 3*D*). The obtained ^15^N-glutamate-α,β,β-d_3_ is subsequently filtered and crystallized to yield a highly pure product with a recovery efficiency of 80% relative to the input α-ketoglutarate-β,β-d_2_.

The final step of the glutamine synthesis is a further enzymatic reaction by glutamine synthetase, which converts ^15^N-glutamate-α,β,β-d_3_ and ^15^N-ammonium to ^15^N_2_-glutamine-α,β,β-d_3_ driven by ATP hydrolysis (Fig. 3, *A*, *D*, and *E*). Again, the reaction mixture is subsequently filtered to remove the enzyme and any precipitants. As judged by the intensity of the residual glutamate ^1^H^γ^ resonance of the final product (Fig. 3*E*), the conversion efficiency from glutamate to glutamine is more than 90%. The produced glutamine shows no proton resonances at the α and β positions, consistent with their deuteration, but two distinct proton resonances corresponding to the diastereotopic γ positions.

It has been reported that the presence of ATP and ADP in the growth medium may be toxic to mammalian cells (14). The filtered final reaction mixture contains ∼60 mM ^15^N_2_-glutamine-α,β,β-d_3_ and ∼120 mM ATP/ADP. The ^15^N_2_-glutamine-α,β,β-d_3_ can be easily separated from the ATP/ADP using inexpensive cation beads, which capture glutamine in a pH-dependent manner (Fig. S5). A comparison of Abl expression obtained with filtered-only ^15^N_2_-glutamine-α,β,β-d_3_, ^15^N_2_-glutamine-α,β,β-d_3_ separated from ATP/ADP by cation beads, or commercially obtained ^15^N_2_-glutamine showed no difference in expression levels (Fig. 3*F*). Thus, the presence of ATP/ADP does not strongly affect insect cell expression. However, the presence of ATP/ADP may be more problematic for other expression systems.

### Production of selectively ^15^N/^2^H-labeled β_1_AR using synthesized ^15^N_2_-glutamine-α,β,β-d_3_

**Figure 4:**
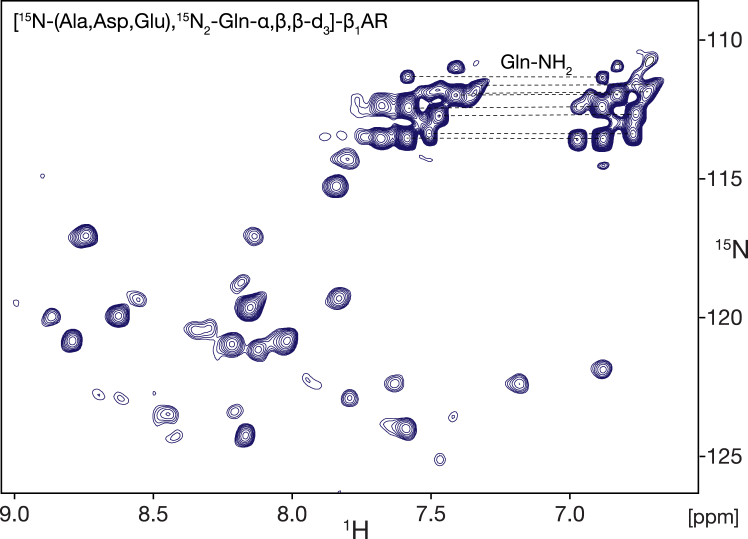
^**1**^**H-**^**15**^**N TROSY NMR spectrum of selectively isotope-labeled β**_**1**_**AR using**^**15**^**N**_**2**_**-glutamine-α,β,β-d**_**3**_**.** β_1_AR was expressed in insect cells grown on a medium containing 250 mg/l synthesized ^15^N_2_-glutamine-α,β,β-d_3_ (filtered only) and 6 g/l commercial unlabeled yeast extract. The receptor is in its active conformation in a ternary complex with the agonist isoprenaline and the G protein mimicking nanobody Nb80 (29). Due to nitrogen scrambling from glutamine, resonances are detected for glutamine, alanine, aspartate and glutamate ^1^H-^15^N moieties. β_1_AR, β_1_-adrenergic receptor; NMR, nuclear magnetic resonance; TROSY, transverse relaxation optimized spectroscopy.

Taking advantage of the identified glutamine nitrogen scrambling routes in insect cells, any protein can be selectively labeled in alanine, aspartate, glutamate, and glutamine from the input of labeled glutamine. We show here this particular type of selective labeling for the β_1_-adrenergic G protein-coupled receptor that we used in previous NMR studies (28, 29, 30, 31). β_1_AR was expressed in 4 L of insect cell growth medium supplemented with 0.25 g/l synthesized ^15^N_2_-glutamine-α,β,β-d_3_ yielding 0.8 mg/l purified, detergent-solubilized receptor. This yield is identical to the yield in standard isotope-labeling medium. The ^1^H-^15^N transverse relaxation optimized spectrum (TROSY) (Fig. 4) of this selectively labeled β_1_AR in the active state complexed with the agonist isoprenaline and the G protein-mimicking nanobody Nb80 (32) shows approximately 60 well resolved resonances out of a total of 72 resonances expected for the ^15^N-labeled alanine, aspartate, glutamate, and glutamine backbone and sidechain moieties. The good resolution indicates that this very simple and selective labeling approach may be useful for obtaining assignments and other information for a considerable part of amino acids in large proteins. The sparseness of the spectrum is also very well suited for the recently developed nanobody GPS–PCS spectral assignment method (31).

## Discussion

### Biochemical mechanisms of glutamine conversion

The importance of glutamine for the metabolism in insect cells and higher eukaryotes stems from its involvement in the TCA cycle in mitochondria (Fig. 2*D*). The TCA cycle is the main powerhouse that drives the production of electron donors, such as NADH and FADH_2_, and the subsequent generation of ATP by oxidative phosphorylation (21). Glutamine fuels the TCA cycle *via* glutaminolysis, also known as glutamine-dependent anaplerosis, which produces α-ketoglutarate. Within the last decade, it has become increasingly clear that glutaminolysis is of high importance in cancer cells, where glutamine provides the main energy to drive the TCA cycle and to generate ATP (33, 34). An apparently similar effect has been reported in the insect cell–baculovirus expression system after viral infection, where glutamine rather than glucose is used to satisfy the energy demand as evidenced by oxygen consumption (19). The strong (60–70%) ^15^N scrambling from ^15^N_2_-glutamine to alanine, aspartate, and glutamate in our experiments shows the high level of glutaminolysis in insect cells after baculovirus infection and completely agrees with this mechanism. During cell growth, ^15^N_2_-glutamine in the medium is transported into the cytoplasm and mitochondrial matrix by glutamine transporters (23). Within the mitochondria, the ^15^N_2_-glutamine is first converted to ^15^N-glutamate by glutaminase or glutamate synthase (Fig. 2*D*) (35, 36).

Glutamate is then deaminated *via* GDH as well as alanine transaminase (AlaAT) and aspartate transaminase (AspAT), and the reaction product α-ketoglutarate enters the TCA cycle for further oxidation and energy production (Fig. 2*D*). GDH splits ammonia from ^15^N-glutamate to form α-ketoglutarate, whereas AlaAT and AspAT transfer the ^15^N-amine group from ^15^N-glutamate to ^15^N-alanine or ^15^N-aspartate, respectively. Alanine is a common metabolic by-product in cultured insect cells (37). AlaAT transfers the ^15^N-amine group from ^15^N-glutamate to pyruvate to form ^15^N-alanine and α-ketoglutarate. In contrast to the direct conversion of glutamate to α-ketoglutarate by GDH, the AlaAT reaction does not produce ammonia, which may be toxic to cells (38), but stores the nitrogen in the alanine amide. AspAT is a highly active enzyme involved in the glutamine metabolism of insect cells (18). It transfers the ^15^N-amine from ^15^N-glutamate to oxaloacetate, thereby producing ^15^N-aspartate and α-ketoglutarate. This may function as a shortcut of the TCA cycle to create upstream α-ketoglutarate by consuming the downstream oxaloacetate.

One full turn of the TCA cycle produces 3 NADH, 1 FADH_2_, and 1 GTP molecules. Subsequent reaction steps by the electron transport chain and oxidative phosphorylation at the inner mitochondrial membrane (21), as well as by the nucleoside-diphosphate kinase produce ∼10 molecules of ATP (assuming a yield of ∼2.5 per NADH, ∼1.5 per FADH_2_, and one per GTP (39)). The shortcut of the TCA cycle by AspAT produces 1 NADH molecule less and accordingly ∼7.5 ATP molecules, *i.e.* still 75% of the full cycle. These mechanisms explain how glutamine can effectively satisfy the high energy demand of insect cells after baculovirus infection. The underlying very strong scrambling of nitrogen from glutamine to alanine, aspartate, and glutamate is made visible by tracing the fate of the glutamine ^15^N atoms in our experiments.

### Isotope labeling

The observed strong nitrogen scrambling from glutamine to alanine, aspartate, and glutamate indicates that the presence of unlabeled glutamine in the growth medium will substantially reduce ^15^N-labeling and subsequently the NMR signals for these three amino acids, if their labeling is attempted by supplementing respective ^15^N-labeled amino acids. On the other hand, growth on a medium that contains ^15^N_2_-labeled glutamine constitutes a simple method for ^15^N-labeling for alanine, aspartate, and glutamate. In the used set-up with ∼200 mg/l unlabeled alanine and glutamate and ∼70 mg/l unlabeled aspartate provided by the yeast extract (Fig. 2*B*), 63 to 67% ^15^N-labeling relative to glutamine was achieved from the ^15^N_2_-glutamine scrambling (Fig. 2*C*). The absolute ^15^N-labeling can be estimated from the mass increase of Abl kinase of 41.9 Da by the ^15^N_2_-glutamine supplementation (Table S1). The provided ^15^N atoms are distributed across glutamine, alanine, aspartate, and glutamate (18, 10, 14, 29 N atoms, respectively) according to the ratios given in Figure 2*C*, which yields an estimate for the absolute ^15^N-labeling of ∼71% for glutamine and ∼46% for alanine, aspartate, and glutamate, respectively. A further increase in the ^15^N-labeling should be achievable for the latter amino acids by reducing the supplementation of their unlabeled forms in the medium to minimal levels. Interestingly, the reduction of glutamine labeling to 71% relative to the simple isotope dilution of 85% shows that ∼14% of ^15^N glutamine is replaced by newly synthesized ^14^N glutamine in the cell. Apparently, this latter process is not very efficient.

The devised method for glutamine deuteration does not comprise the γ positions as they do not exchange in α-ketoglutarate at high pD. However, this is only a minor drawback as the dipolar interactions of the γ protons to the amide groups are considerably weaker than those of the α and β protons. Thus, their contributions to the amide proton transverse and longitudinal relaxation will be small in NMR experiments with ^1^H-^15^N observation.

### Supplementation of ^15^N_2_-glutamine by other methods

Several reports (8, 10, 40) have suggested that ^15^N_2_-glutamine could be replaced in the insect cell medium by ^15^NH_4_Cl under the assumption that glutamine can be synthesized from ammonium and glutamate *via* glutamine synthetase. However, a test showed that replacing the 1 g/l (7 mM) ^15^N_2_-glutamine by 5 mM ^15^NH_4_Cl significantly lowered (∼6 times) the expression yields for both GFP and Abl kinase (Fig. S6). This finding is in agreement with the observed low efficiency (∼14%) of glutamine synthesis in the cell mentioned above. A further strategy to supply the necessary glutamine by co-expression of glutamine synthetase has been reported for mammalian cells (14, 41). This may also be possible in insect cells but has not yet been tested. In comparison, the direct supplementation of glutamine is highly practical and efficient for all systems.

### Costs

The described procedure for the large-scale production of ^15^N_2_-glutamine-α,β,β-d_3_ (or ^15^N_2_-glutamine) is very robust and economical. The total costs amount to only ∼119 Euro/g for ^15^N_2_-glutamine-α,β,β-d_3_ and ∼100 Euro/g for ^15^N_2_-glutamine (Table S3). This is significantly less expensive than commercially available ^15^N_2_-glutamine-d_5_ (∼5000 Euro/g) and ^15^N_2_-glutamine (1500–3000 Euro/g) and makes labeled glutamine supplementation easily affordable. Even further substantial reductions in costs may be achieved by producing the enzymes GDH and FDH instead of using a commercial supply.

## Conclusion

Our results show how glutaminolysis is used in insect cells to satisfy the increased energy demand in insect cells under baculovirus infection, which explains the absolute requirement of glutamine for efficient protein expression and isotope labeling. The supply of isotope-labeled glutamine is necessary not only for the effective labeling of glutamine but also for alanine, aspartate, and glutamate. It is expected that the inexpensive isotope-labeled glutamine provided by the described procedure will facilitate the high-resolution NMR analysis of complex proteins expressed in insect cells and other higher eukaryotic cells.

## Experimental procedures

Detailed materials and methods including protein constructs, preparation of yeast extracts, protein expression and purification, production of isotope-labeled glutamine, GFP fluorescence measurement, Western blot analysis, mass spectrometry, and NMR experiments are provided in the supporting information.

## Data availability

All data are included in the manuscript.

## Supporting information

This article contains supporting information (42, 43).

## Conflict of interest

The authors declare no conflict of interest with the contents of this article.
