# Supplementary material for: The key role of glutamine for protein expression and isotopic labeling in insect cells
Source: J Biol Chem. 2023 Aug 6;299(10):105142. doi: 10.1016/j.jbc.2023.105142 (PMC10556780; doi:10.1016/j.jbc.2023.105142)
Supplement: Supporting Information [file mmc1.pdf]

The key role of glutamine for protein expression and isotopic labeling in insect cells

Feng-Jie Wu[1*], Domenic Kronenberg[1], Ines Hertel[1], Stephan Grzesiek[1*]

[1]Biozentrum, University of Basel, CH-4056 Basel, Switzerland

Supporting Information

*Address correspondence to:
Feng-Jie Wu
Biozentrum, University of Basel, CH-4056 Basel, Switzerland
Email: fengjie.wu@unibas.ch

Stephan Grzesiek
Biozentrum, University of Basel, CH-4056 Basel, Switzerland
Phone: ++41 61 207 2100
Email: Stephan.Grzesiek@unibas.ch

Experimental procedures

Preparation of yeast extracts

^{15}N-labeled and unlabeled yeast extracts were prepared as described previously (1).

Protein constructs

The following protein constructs were used for all analyses: GFP, trGFPuv (2); Abl kinase, His$_6$-TEVsite-GAMDP-hABL$^{S229–S500}$ (isoform 1A numbering) (3, 4); β_1AR, stabilized turkey YY-β_1AR with a C-terminal His$_6$-tag (5).

Small-scale protein expression tests in insect cells

Test of glutamine supplementation

Insect cell growth medium for testing the glutamine dependence was prepared by adding 8 g/L ^{15}N labeled yeast extract (made in-house) and different concentrations of $^{15}N_2$-glutamine (Cambridge isotope laboratories), homemade $^{15}N_2$-glutamine-α,β,β-d_3, or unlabeled $^{14}N_2$-glutamine to ΔSF4 medium (Bioconcept) devoid of yeast extract and amino acids. The osmolarity was adjusted to ~340 mOsm using 5 M NaCl, and the pH was adjusted to 6.3. A further starvation medium to remove unlabeled amino acids was prepared as ΔSF4 medium with its osmolarity adjusted to ~340 mOsm. Before use, all media were sterilized using a 0.22-µm filter.

Sf9 insect cells (Oxford expression technologies) were prepared at a density of 2–4 × 10^6 cells/mL in SF4 insect cell medium containing yeast extract and all amino acids (Bioconcept). Cells were pelleted at 500 g centrifugation for 5 min at room temperature. Subsequently, the supernatant was removed and cells were resuspended gently in the pre-warmed (27 ˚C) starvation medium in a 50-mL TPP® TubeSpin bioreactor tube to reach a final cell density of 2.5 × 10^6 cells/mL. Cells were then incubated in an orbital shaker (25 mm diameter) for 2 h at 250 rpm and 27 ˚C. After starvation, cells were pelleted as before to remove the supernatant. Cells were then gently resuspended with pre-warmed (27 ˚C) glutamine-testing medium to reach again a cell density of 2.5 × 10^6 cells/mL. Baculovirus for production of the respective target protein was then added, and the cells were incubated in an orbital shaker (25 mm diameter) at 250 rpm and 27 ˚C for protein expression. Cells were harvested at 66–72 hours post infection (hpi) for GFP and Abl kinase, and 48 hpi for β_1AR.

Test of ammonium supplementation

For testing the replacement of $^{15}N_2$-glutamine by $^{15}NH_4Cl$, a growth medium was prepared by supplementing 8 g/L ^{15}N labeled yeast extract (made in-house) and 5 mM $^{15}NH_4Cl$ to ΔSF4 medium, with the osmolarity adjusted to ~340 mOsm and the pH adjusted to 6.3. *Sf9* insect cells

were prepared at a density of 2.5 × 10^6 cells/mL and infected with the baculovirus. At 16 hpi, cells were gently centrifuged at 500 g for 5 min at room temperature. The supernatant was removed and cells were resuspended with an identical volume of pre-warmed (27 ˚C) $^{15}NH_4Cl$-containing medium in a 50-mL TPP® TubeSpin bioreactor tube. Cells were then incubated in an orbital shaker (25 mm diameter) at 250 rpm and 27 ˚C for protein expression. Cells were harvested at 66–72 hpi for GFP and Abl kinase.

GFP fluorescence measurement

A 200-µL volume of insect cells expressing GFP was transferred to a black, non-binding Greiner Bio-One 96-well microplate. GFP fluorescence was measured using a synergy H1 hybrid microplate reader (BioTek®) with 485 nm excitation and 528 nm emission wavelengths.

Western blot analysis

For quantifying Abl kinase or β_1AR expression levels a quantitative western blot analysis was carried at room temperature as follows. 10 µL Abl kinase or β_1AR harboring a histidine tag was separated by SDS-PAGE and transferred to a nitrocellulose membrane. Blots were then blocked with 1% BSA in Tris-Buffered Saline-Tween (TBST) for 1 h and then incubated with a monoclonal anti-polyHistidine-Peroxidase antibody (A7058, Sigma) at 1:5000 dilution for 1 h in the dark. Blots were then washed three times for 5 min with TBST, developed with chemiluminescent substrate, and analyzed by chemiluminescence detection.

Intensities of the protein bands on the western blot images were then integrated and quantified using the program Fiji (6).

Mass spectrometry

For determination of the total protein mass and the efficiency of isotope incorporation, purified protein samples were first desalted by solid phase extraction on a C4 reverse phase micro spin column according to the supplier's protocol (The Nest Group). The total protein mass was then determined by ESI-TOF mass spectrometry (Bruker microTOF) applying direct infusion of the prepared proteins. Mass spectra were analyzed by maximum entropy deconvolution. Isotope incorporation was determined relative to the detected mass of the respective unlabeled protein.

For the determination of isotope incorporation into single amino acids of expressed proteins, the proteins were first hydrolyzed by the following procedure. 20 µg protein ($^{15}N_2$-glutamine-labeled or uniformly ^{15}N-labeled Abl kinase) were placed into pyrolyzed (550 °C for 24 h) 0.3-mL glass vials (Chromacol GOLD-Grade, Thermo Scientific) and dried for 15 min in a speedvac. 6 N HCl was incubated by argon gas for 15 mins to remove air. The protein sample

vials were then placed into a 50-mL Pyrex bottle with their positions stabilized by a homemade Teflon holder. The bottom of the Pyrex bottle was covered with 4 mL of the pretreated 6 N HCl. The bottle was then incubated again with argon gas for 15 min to remove air. Thereafter the bottle was closed tightly and incubated for 24 h at 110 °C. After incubation, the bottle was cooled down for 5 min to room temperature and the outer surface of the sample vials was wiped with 100% EtOH before drying them in a speedvac for 5 min. The final hydrolyzed samples were stored at -20 °C.

Quantitative amino acid analysis of yeast extracts or hydrolyzed Abl was carried out as previously described (1, 7). Briefly, 2.7 mg yeast extract or 20 µg hydrolyzed protein were derivatized with phenyl isothiocyanate (PITC) in a 270-µL reaction volume. Unlabeled standards for each amino acid were prepared accordingly. The derivatized phenylthiohydantoin (PTH) amino acids were then separated via HPLC (Thermo Fisher Scientific) on an Eclipse XDB-C18 reverse phase column (Agilent) followed by mass spectrometry (Bruker microTOF) for their identification and quantification of their isotope content.

Production of glutamine synthetase

The synthesized gene of glutamine synthetase (glnA1) from Mycobacterium tuberculosis (Uniprot entry P9WN39) was obtained from GenScript and cloned into a pET-11a vector containing an N-terminal 6×His-tag. The plasmid was transformed into *E. coli* BL21 competent cells. A single colony was then picked for growth of an overnight culture in 100 mL lysogeny broth (LB) medium at 37 °C, 110 rpm using an Infors Multitron incubation shaker. 10-mL volumes of the overnight culture were then inoculated into 4 × 1 L LB medium in 5 L flasks. The cell cultures were grown at 37 °C with 110 rpm shaking speed until the optical density (OD) reached 0.6. Protein expression was induced by addition of 1 mM isopropyl-D-thiogalactopyranoside (IPTG) and carried out for 4 h at 30 °C. Cells were harvested by centrifugation at 5000 g for 20 min.

The pellet was resuspended in lysis buffer (20 mM Tris-HCl pH 8, 100 mM NaCl, 20 mM $MgCl_2$, 0.5 mg/mL lysozyme, and a small spatula of DNase I), and gently stirred at room temperature for 1 h. Cells were then lysed using an ultrasonic cell disruptor (Branson Digital Sonifier®) at 30% power for 10 min of consecutive 10 seconds on/off intervals. The supernatant was separated from the cell debris by centrifugation at 10000 g for 20 min at 4 °C and subsequently applied to 8 ml Ni-NTA beads pre-equilibrated with base buffer (20 mM Tris-HCl pH 8, 100 mM NaCl, 20 mM $MgCl_2$, and 10 mM imidazole). The beads were then washed with 80 mL base buffer and eluted with 30 mL elution buffer (base buffer supplemented with 300 mM imidazole). The eluate was concentrated to 4 mL and applied to a HiLoad 16/600 Superdex

200 gel-filtration column (GE Healthcare) pre-equilibrated with 50 mM sodium phosphate pH 7.5, 100 mM NaCl, and 20 mM $MgCl_2$. The size exclusion fractions containing glutamine synthetase were pooled and concentrated in a 100 kDa MWCO centrifugal filter unit (Millipore) to a final concentration of 5 mg/mL.

Large-scale expression and purification isotope-labeled Abl kinase

For expression of isotope-labeled Abl kinase, 400 mL labeling medium was prepared by supplementing 8 g/L ^{15}N-labeled or ^{14}N-unlabeled yeast extract (both made in-house) and 250 mg/L $^{15}N_2$-glutamine (Cambridge Isotope Laboratories) to ΔSF4 medium, with the osmolarity adjusted to ~340 mOsm and the pH adjusted to 6.3. The medium was sterilized through a 0.22 µm filter before use. For medium exchange, *sf9* insect cells grown in SF4 medium to a density of 2.5–3.0 × 10^6 cells/mL were centrifuged at 500 g for 10 min at room temperature, and the cell pellet was gently resuspended in 200 mL pre-warmed (27 ˚C) starvation medium (ΔSF4, see above) to a density of 5.0 × 10^6 cells/mL. After 2 h starvation, cells were pelleted again at 500 g for 10 min at room temperature. The supernatant was discarded and the cells were resuspended in 400 mL pre-warmed (27 ˚C) labeling medium. Abl kinase baculovirus and the ATP-site inhibitor imatinib (17 µM final concentration) were added to the cells, and the expression was started in an orbital shaker at 180 rpm (50 mm diameter) and 27 ˚C. Cells were harvested at 66 hpi by centrifugation and the cell pellet was resuspended in lysis buffer (20 mM Tris-HCl, 100 mM NaCl, pH 8) supplemented with one tablet of cOmplete™, EDTA-free protease inhibitor cocktail (Roche). The cell suspension was then frozen in a 50-mL Falcon® tube using liquid nitrogen and stored in a -80 ˚C freezer until further use. The subsequent purification of Abl kinase was carried out as previously described (4).

Large-scale expression and purification of isotope-labeled β_1AR

4 L labeling medium was prepared by supplementing 6 g/L unlabeled yeast extract (Bioconcept) and 250 mg/L (1 g total) homemade $^{15}N_2$-glutamine-α,β,β-d_3 to ΔSF4 medium, with osmolarity and pH adjusted to ~340 mOsm and 6.3, respectively. The medium was sterilized through a 0.22 µm filter before use.

For receptor expression, 4 L of *sf9* insect cells were prepared in unlabeled SF4 medium at a density of 3.0 × 10^6 cells/mL, which were distributed over 8 600-mL TPP® TubeSpin bioreactor bottles. The cells in each bottle were then centrifuged at 500 g for 10 min at room temperature and the SF4 medium in the supernatant was removed. The pelleted cells were then gently resuspended in the same bottle with 500 mL pre-warmed (27 ˚C) labeling medium. Thereafter

10 mL/L high-titer YY-β_1AR baculovirus was added and cells were incubated in an orbital shaker (50 mm diameter) at 180 rpm and 27 °C for 48 h before harvesting.

Receptor purification was carried out as described previously (8).

NMR experiments

NMR samples of 270-μL volume 208 μM $^{15}N_2$-glutamine-labeled or 144 μM uniformly ^{15}N-labeled Abl kinase were prepared in 20 mM Bis-Tris (pH 6.5), 150 mM NaCl, 2 mM EDTA, 3 mM TCEP, 0.02% NaN_3, 5% D_2O and placed in 5-mm Shigemi microtubes. NMR experiments were performed on a Bruker AVANCE 900 MHz spectrometer equipped with a TCI cryogenic probe at 298 K. 2D ^1H-^{15}N HSQC spectra were recorded as described previously (8) as 168 (^{15}N) × 2048 (^1H) complex points and acquisition times of 30 ms (^{15}N) and 80 ms (^1H), respectively. The total experimental times were 7 h for both $^{15}N_2$-glutamine labeled and uniformly ^{15}N-labeled Abl kinase.

A 270-μL NMR sample of 150 μM $^{15}N_2$-glutamine-α,β,β-d_3-labeled β_1AR was prepared in 20 mM Tris (pH 7.5), 100 mM NaCl, 0.1% DM, 5% D_2O and placed in a 5-mm Shigemi microtube. NMR experiments were performed on a Bruker AVANCE 900 MHz spectrometer equipped with a TCI cryogenic probe at 304 K. The ^1H-^{15}N TROSY spectrum was recorded as 80 (^{15}N) × 1024 (^1H) complex points with acquisition times of 16 ms (^{15}N) and 43 ms (^1H), respectively and a total experimental time of 24 h.

Figures

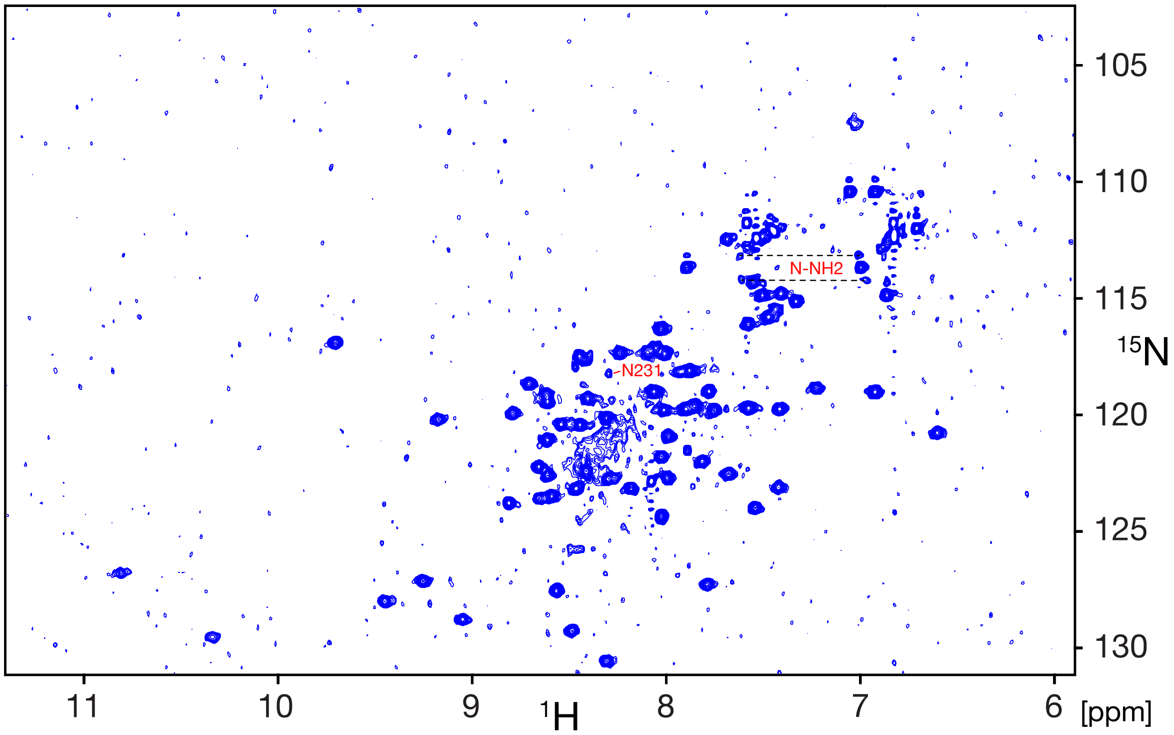

Figure S1. ^{1}H-^{15}N HSQC NMR spectrum of Abl kinase expressed in insect cell medium containing 250 mg/L $^{15}N_2$-glutamine and 8 g/L unlabeled yeast extract. The spectrum is identical to the one in Figure 2A, but displayed at the noise level threshold (3.14 times lower). In both Figures, the contour level spacing corresponds to a factor 1.25.

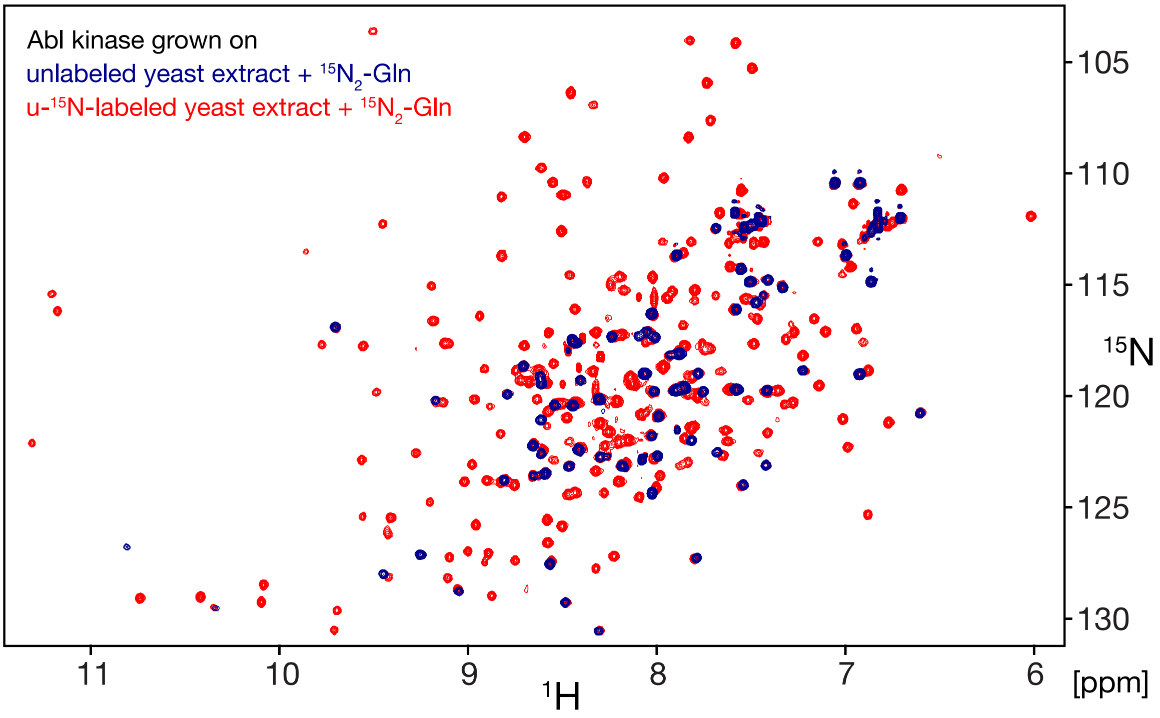

Figure S2. Superimposed ^{1}H-^{15}N HSQC NMR spectra of Abl kinase expressed in insect cell media containing 250 mg/L $^{15}N_2$-glutamine and 8 g/L unlabeled yeast extract (dark blue) or 250 mg/L $^{15}N_2$-glutamine and 8 g/L ^{15}N-labeled yeast extract (red).

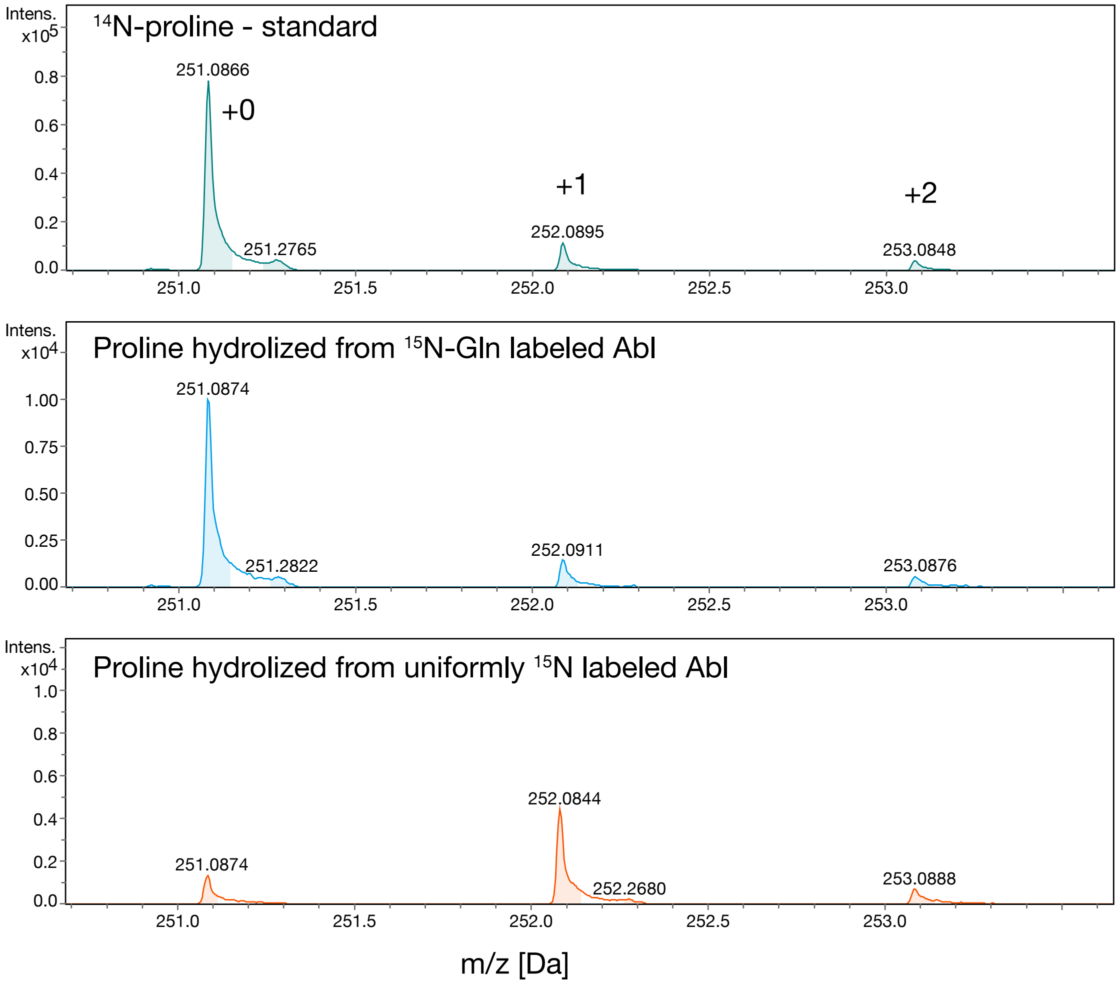

Figure S3. Control of ^{15}N scrambling from $^{15}N_2$-glutamine to proline during Abl expression by HPLC mass spectrometry of the amino acids from hydrolyzed Abl. Top: control of unlabeled proline standard. Middle: proline hydrolyzed from Abl expressed with 250 mg/L $^{15}N_2$-glutamine supplementation to 8 g/L unlabeled yeast extract. Bottom: proline hydrolyzed from uniformly ^{15}N-labeled Abl. The difference in the intensity ratios of the proline +1 peak relative to the proline +0 peak between the $^{15}N_2$-glutamine labeled Abl (middle) and the unlabeled proline standard (top) is less than 0.2%.

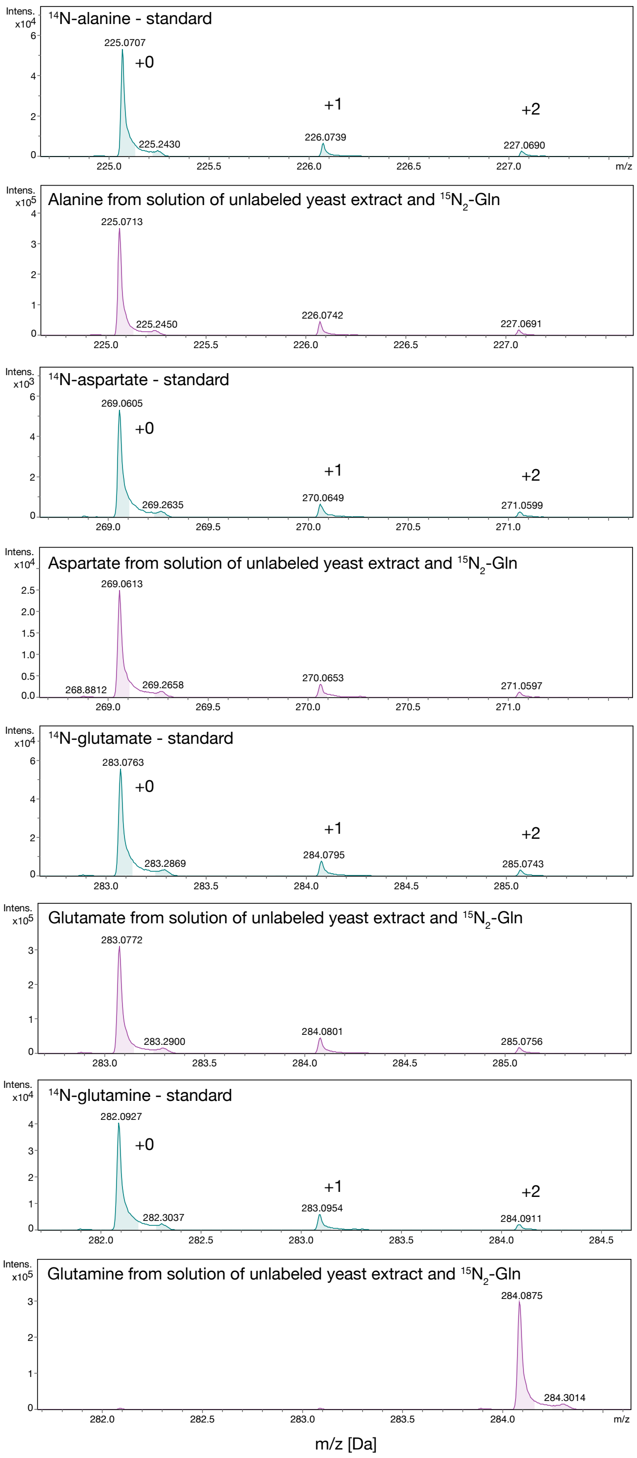

Figure S4. HPLC mass spectrometry test of ^{15}N scrambling from $^{15}N_2$-glutamine to alanine, aspartate, and glutamate within mixture of unlabeled yeast extract and $^{15}N_2$-glutamine. A 500-µL solution of 8 g/L unlabeled yeast extract mixed with 250 mg/L $^{15}N_2$-glutamine (pH 6.3) was incubated at 27 ˚C for 24 h and then analyzed by HPLC mass spectrometry. The differences in the intensity ratios of the +1 peak relative to the +0 peak for alanine, aspartate or glutamate between the incubated solution mixture and the unlabeled standard are less than 0.5%. For glutamine, there is no detectable, unlabeled glutamine at the +0 position and only $^{15}N_2$-labeled at the +2 position, indicating that no unlabeled glutamine is present in the yeast extract.

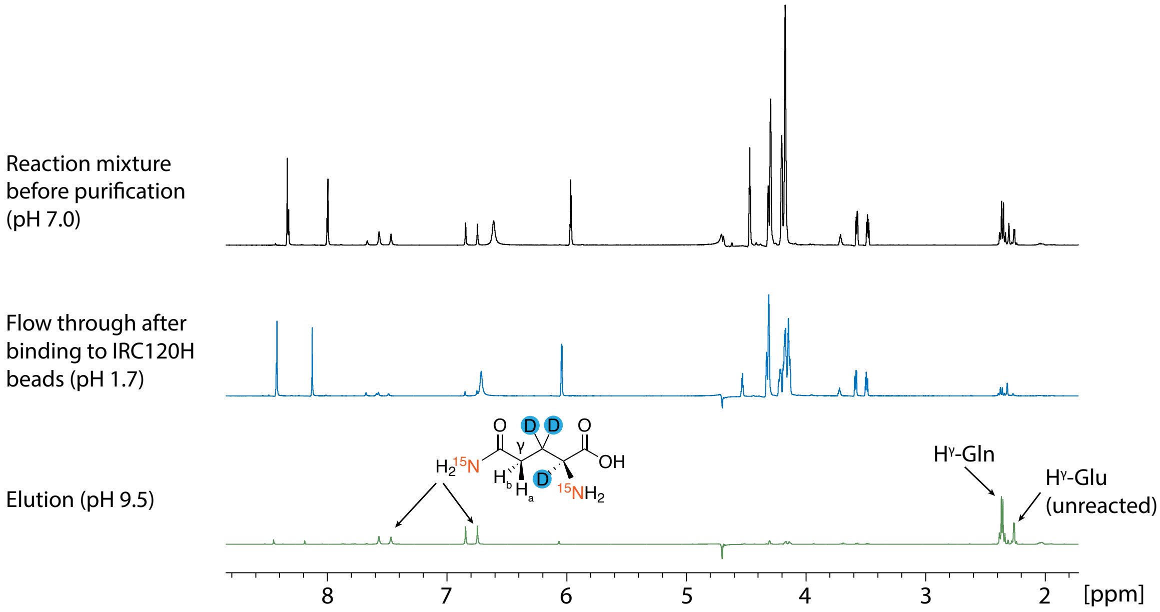

Figure S5. ^{1}H NMR quality control of the purification of synthesized $^{15}N_2$-glutamine-α,β,β-d_3 using IRC120H beads via pH adjustment.

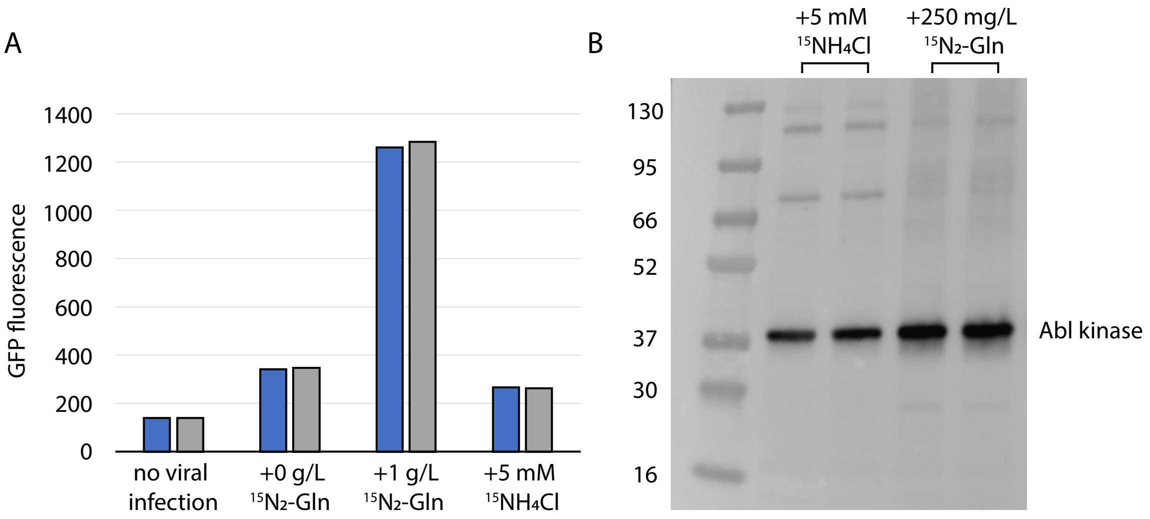

Figure S6. Comparison of insect cell protein expression level using either $^{15}N_2$-glutamine or $^{15}NH_4Cl$ supplementation in the yeast extract-based growth medium. (A) GFP quantified by fluorescence. (B) Abl kinase quantified by anti-His western blot. Molecular weight markers in are annotated in kDa on the left.

Tables

Table S1 ^{15}N incorporation of proteins expressed in insect cells using a medium containing 8 g/L ^{15}N-labeled yeast extract[a] supplemented with either 1 g/L $^{14}N_2$- or $^{15}N_2$-glutamine

Protein		$^{14}N_2$-glutamine	$^{15}N_2$-glutamine	difference
GFP	mass difference to unlabeled[b]	234.0	261.8	27.8
	^{15}N incorporation[c]	76.7%	85.8%	9.1%
Abl kinase	mass difference to unlabeled	300.7	342.6	41.9
	^{15}N incorporation	74.8%	85.2%	10.4%

[a]the ^{15}N incorporation of the used yeast extract was determined as 95%.

[b]in Da. Determined by mass spectrometry from the total detected mass of the expressed protein. The detected masses for proteins expressed in unlabeled medium were 25583.2 Da for GFP and 34893.5 Da for Abl kinase, respectively.

[c]the total number of nitrogen atoms is 305 for GFP and 402 for Abl kinase, respectively.

Table S2 Protocol for the cost-effective, large-scale production of $^{15}N_2$-glutamine-α,β,β-d_3

The following protocol would generate a total of 11.5 grams of $^{15}N_2$-glutamine-α,β,β-d_3 from 20 grams α-ketoglutarate, if all material obtained in the intermediate steps is used.

Preparation of α-ketoglutarate-β,β-d_2 from α-ketoglutarate

1. Dissolve 20 g α-ketoglutarate (Carl Roth) in 200 mL 99.8% D_2O.
2. Adjust pD of solution to 10 using NaOD.
3. Incubate for 16 h at 45 °C.
4. Check reaction by ^{1}H-NMR. Stop reaction when protons at β position are completely exchanged with deuterium. Unwanted byproducts will appear if the reaction is carried out for too long (>20 h).
5. Change pD of solution to 7 by HCl.
6. Lyophilize.

A total of 15.6 grams lyophilized α-ketoglutarate-β,β-d_2 was obtained, corresponding to 78% recovery efficiency.

Enzymatic synthesis of ^{15}N-glutamate-α,β,β-d3 from α-ketoglutarate-β,β-d2

Prepare glutamate reaction solution

1. Prepare 300 mL reaction solution in Milli-Q® water by dissolving
   - 8.76 g (200 mM) α-ketoglutarate-β,β-d_2
   - 3.27 g (200 mM) $^{15}NH_4Cl$ (Cambridge Isotope Laboratories)
   - 8.16 g (400 mM) d-sodium formate (Biosynth)
   - 200 mg (1 mM) NADH (Sigma)
2. Adjust pH to 8 using NaOH
3. Sterile filter and store at 4 °C.

Enzymatic glutamate synthesis (enzymes are recycled twice by filtering)

1. Take 100 mL of glutamate reaction solution and add
   - 2000 U (20 U/mL) glutamate dehydrogenase (GDH, Roche)
   - 100 U (1 U/mL) formate dehydrogenase (FDH, Roche)
2. Run reaction at 25 °C in a flask under shaking or in a beaker under stirring (about 6 hours in our hands).
3. Follow reaction by ^{1}H-NMR or polarimetric analysis at 589 nm.
4. Upon reaction completion (e.g. shift of $^1H^\gamma$ resonances), filter mixture through a 10 kDa cut-off ultrafiltration disc in an Amicon® stirred cell (both Millipore) to separate enzymes in the filtrate for recycling. Keep permeate for crystallization.
5. Repeat reaction steps 2–4 twice by adding recycled enzymes to two further 100-mL batches of glutamate reaction solution.

Crystallization

1. Combine permeates from all reaction repetitions.
2. Adjust pH of combined permeate to 3.2.
3. Cool solution to 4 °C and let crystalize overnight.
4. Separate crystals from solution by filtration (glass frit, fine porosity). Keep permeate.
5. Wash crystals with ice-cold Milli-Q water.
6. Dry crystals in desiccator.
7. Perform another crystallization round with the permeate of step 4 using the obtained ^{15}N-glutamate-α,β,β-d_3 crystals as seeds.
8. Dry the crystals under vacuum to obtain glutamate in powder form.
9. Analyze purity and isotope incorporation ratio by NMR or LC/MS.

In total, 7.2 g ^{15}N-glutamate-α,β,β-d_3 was obtained from the initial 8.76 g α-ketoglutarate-β,β-d_2, corresponding to a molar recovery efficiency of 80%.

Enzymatic synthesis of $^{15}N_2$-glutamine-α,β,β-d_3 from ^{15}N-glutamate-α,β,β-d_3

1. Prepare 500 mL of reaction mixture in Milli-Q® water by dissolving
   - 5 g (67 mM) ^{15}N-glutamate-α,β,β-d_3
   - 1.8 g (67 mM) $^{15}NH_4Cl$ (Cambridge Isotope Laboratories)
   - 33 g (120 mM) ATP-2Na (Carl Roth)
   - 9.5 g (93 mM) $MgCl_2 \cdot 6\ H_2O$ (Carl Roth)
   - 10–20 mg glutamine synthetase (made in-house)
2. Adjust pH to 7.8 and sterile filter.
3. Run reaction at 37 °C for 24–48 h [a white precipitate of presumably $MgHPO_4$ or $Mg_3(PO4)_2$ may start to appear if reaction runs longer than 48 h].
4. As the pH drops during the reaction from the release of protons, maintain the pH either manually or automatically (bioreactor) in the range 7.2–7.8 to keep the enzyme in its active state (the activity drops significantly below pH 6.5).
5. Monitor the reaction by ^1H-NMR, thin-layer chromatography, or LC/MS.
6. Stop the reaction, when >90% ^{15}N-glutamate-α,β,β-d_3 is converted to $^{15}N_2$-glutamine-α,β,β-d_3.
7. Clear reaction mixture through a 0.22 μm filter membrane to remove any precipitates.
8. Filter cleared reaction mixture through a 10-kDa cut-off ultrafiltration disc in an Amicon® stirred cell (both Millipore)
9. Keep filtration permeate containing the synthesized $^{15}N_2$-glutamine-α,β,β-d_3.
10. If further purification is not required, adjust reaction mixture to pH 7 and reduce volume to 25–50 mL using an evaporator.
11. Determine concentration of $^{15}N_2$-glutamine-α,β,β-d_3 by ^1H-NMR, thin-layer chromatography, or LC/MS.

As a final yield, 4.5 g $^{15}N_2$-glutamine-α,β,β-d_3 were obtained from 5 g ^{15}N-glutamate-α,β,β-d_3, corresponding to a molar recovery efficiency of 90%.

Further purification of $^{15}N_2$-glutamine-α,β,β-d_3 (required for ATP/ADP-sensitive cells)

1. Wash 300 g Amberlite® IRC120H ion exchange beads (Sigma) three times with 300 mL Milli-Q® water.
2. Mix 500 mL final (~4.5 g) $^{15}N_2$-glutamine-α,β,β-d_3 reaction mixture with the washed IRC120H beads in a 1-L beaker.
3. Adjust pH to 1–2 using HCl and gently stir for 10 min.
4. Wait briefly until beads are completely settled. Remove supernatant.
5. Add 300 mL Milli-Q® water to the beads.
6. Adjust pH to 2–3 using NaOH and gently stir for 5 min.
7. Wait briefly until beads are completely settled. Remove supernatant.
8. Repeat washing steps 5-7 for two further times.
9. For elution, add 150 mL Milli-Q® water to the beads.
10. Adjust pH carefully to 9–10 using 10 M NaOH and gently stir for 15 min.
11. Wait briefly until beads are completely settled. Collect eluted supernatant.
12. Repeat elution procedure once again.
13. Combine elutions and adjust final pH to 7 using NaOH. Condense volume to 25–50 mL using an evaporator.
14. Determine concentration of $^{15}N_2$-glutamine-α,β,β-d_3 by ^1H-NMR, thin-layer chromatography, or LC/MS.

Table S3 Cost analysis for the production of 1 g isotope-labeled glutamine

	Amount	$^{15}N_2$-glutamine[a]	$^{15}N_2$-glutamine-α,β,β-d_3[a]
**Glutamate synthesis (80% molar efficiency from α-ketoglutarate)**			
α-ketoglutarate	1.7 g	1.2	1.2
D_2O	17 mL	-	10.7
$^{15}NH_4Cl$	0.5 g	15.1	15.1
sodium formate	1.3 g	0.8	9.3
NADH	30.7 mg	2.3	2.3
GDH (recycled twice)	308.6 U	10.0	10.0
FDH (recycled twice)	15.4 U	31.9	31.9
**Glutamine synthesis (90% molar efficiency from glutamate)**			
$^{15}NH_4Cl$	0.4 g	11.0	11.0
ATP-Na_2	7.3 g	21.3	21.3
$MgCl_2 \cdot 6\ H_2O$	2.1 g	0.1	0.1
glutamine synthetase[b]	10–20 mg	-	-
**Additional glutamine purification (optional)**			
IRC120H beads	60 g	6.42	6.42
Total		100.4	119.4

[a]cost in Euro.
[b]made in-house

References

1. Opitz, C., Isogai, S., and Grzesiek, S. (2015) An economic approach to efficient isotope labeling in insect cells using homemade 15N-, 13C- and 2H-labeled yeast extracts. *J. Biomol. NMR*. **62**, 373–385
2. Khan, F., Stott, K., and Jackson, S. (2003) 1H, 15N and 13C backbone assignment of the green fluorescent protein (GFP). *J. Biomol. NMR*. **26**, 281–282
3. Strauss, A., Bitsch, F., Fendrich, G., Graff, P., Knecht, R., Meyhack, B., and Jahnke, W. (2005) Efficient uniform isotope labeling of Abl kinase expressed in Baculovirus-infected insect cells. *J. Biomol. NMR*. **31**, 343–349
4. Vajpai, N., Strauss, A., Fendrich, G., Cowan-Jacob, S. W., Manley, P. W., Grzesiek, S., and Jahnke, W. (2008) Solution Conformations and Dynamics of ABL Kinase-Inhibitor Complexes Determined by NMR Substantiate the Different Binding Modes of Imatinib/Nilotinib and Dasatinib. *J. Biol. Chem.* **283**, 18292–18302
5. Grahl, A., Abiko, L. A., Isogai, S., Sharpe, T., and Grzesiek, S. (2020) A high-resolution description of β1-adrenergic receptor functional dynamics and allosteric coupling from backbone NMR. *Nat. Commun.* **11**, 2216
6. Schindelin, J., Arganda-Carreras, I., Frise, E., Kaynig, V., Longair, M., Pietzsch, T., Preibisch, S., Rueden, C., Saalfeld, S., Schmid, B., Tinevez, J.-Y., White, D. J., Hartenstein, V., Eliceiri, K., Tomancak, P., and Cardona, A. (2012) Fiji: an open-source platform for biological-image analysis. *Nat. Methods*. **9**, 676–682
7. Franke, B., Opitz, C., Isogai, S., Grahl, A., Delgado, L., Gossert, A. D., and Grzesiek, S. (2018) Production of isotope-labeled proteins in insect cells for NMR. *J. Biomol. NMR*. **71**, 173–184
8. Isogai, S., Deupi, X., Opitz, C., Heydenreich, F. M., Tsai, C.-J., Brueckner, F., Schertler, G. F. X., Veprintsev, D. B., and Grzesiek, S. (2016) Backbone NMR reveals allosteric signal transduction networks in the β1-adrenergic receptor. *Nature*. **530**, 237–241
